# Effects of nimodipine on postoperative delirium in elderly under general anesthesia: A prospective, randomized, controlled clinical trial

**DOI:** 10.1097/MD.0000000000006849

**Published:** 2017-05-12

**Authors:** Ya-Nan Li, Qi Zhang, Chun-Ping Yin, Yang-Yang Guo, Shu-Ping Huo, Liang Wang, Qiu-Jun Wang

**Affiliations:** Department of Anesthesiology, The Third Hospital of Hebei Medical University, Hebei, China.

**Keywords:** aged, brain, delirium, metabolism, nimodipine

## Abstract

Nimodipine is a clinical commonly used calcium antagonistscan lowering the apoptosis rate of hippocampal neuron to reduce the incidence of postoperative cognitive dysfunction (POCD). This study was designed to evaluate the effects of nimodipine on postoperative delirium in elderly under general anesthesia.

Sixty patients shceduced spine surgery under general anesthesia were randomly assigned into 2 groups using a random number table: control group (Group C) and nimodipine group (Group N). In Group N, nimodipine 7.5 μg/(kg × h) was injected continually 30 minutes before anesthesia induction, while the equal volume of normal saline was given in Group C. At 0 minute before injection, 0 minute after tracheal intubation, 1 hour after skin incision and surgery completed (T_1–4_), blood samples were taken from the radial artery and jugular bulb for blood gas analysis. Cerebral oxygen metabolism-related indicators were calculated at the same time. Concentration of S100β and glial fibrillary acidic protein (GFAP) were tested by ELISA. The incidence of postoperative delirium within 7 days after surgery was recorded.

Cerebral oxygen metabolism-related indicators fluctuationed in the normal range in 2 groups at different time points and the difference were not statistically significant. Compared with Group C, S100β and GFAP decreased and incidence of postoperative delirium reduced at T_3–4_ in Group N, the difference was statistically significant (*P*<.05).

The present study suggests that nimodipine can reduce the development of postoperative delirium in elderly patients under general anesthesia, the reduction of brain injury and improvement of cerebral oxygen metabolism may be involved in the mechanism.

## 1 Introduction

Postoperative delirium is a major clinical syndrome in geriatric surgical patients with the characteristics of prolonged hospitalization, increased risk of postoperative complications, and significant mortality.^[1,2]^ It occurs at an incidence rate of 15% to 35% in the patients over the age of 65 years within 2 weeks after surgery.^[3]^ Therefore, finding an appropriate preventive therapy to postoperative delirium is important. Although the pathogenesis of postoperative delirium remains to be clarified, many studies have identified cerebral damage as a major risk factor for postoperative delirium.^[4]^ General anesthesia is an important anesthesia method characterized by unconsciousness, analgesia, and muscle relaxation, and it is suitable for large trauma, wide range, and complex operation. Saniova et al^[5]^ reported that general anesthesia is one of the independent risk factors of postoperative delirium.

Nimodipine, a clinical commonly used 1, 4-two hydrogen pyridine type calcium antagonists, could penetrate the blood–brain barrier easily and inhibit calcium channel on the nerve cells and the brain microvascular endothelial cell membrane specifically. It can expand the cerebral blood vessels, improve the cerebral circulation, and protect the brain.^[6,7]^ Studies have shown that nimodipine could improve cognition in adult mice by reducing neuronal damage^[8]^ and our previous research found pretreatment with nimodipine can prevent postoperative cognitive dysfunction (POCD) on aged rats by reducing hippocampal apoptosis rate.^[9]^ However, its clinical effect remains to be discussed.

The purpose of this study is to evaluate the effects of nimodipine on postoperative delirium in elderly under general anesthesia. The results of this study could provide reference for clinical practice.

## 2 Materials and methods

### 2.1 Ethics statement

The present study has been performed with the approval of the ethics committee of the Third Hospital of Hebei Medical University and is in compliance with the Helsinki Declaration. The informed consents of the study were collected from all the candidate subjects.

### 2.2 Patients and inclusion criteria

We collected 60 patients (7 patients were lost because of noncooperation and 4 patients were lost by not receiving operation) shceduced spine surgery under general anesthesia from September 2016 to February 2017 in the Third Hospital of Hebei Medical University, American Society of Anesthesiologists’ physical status classes I and II. Patients have no obvious abnormality of heart, lung, liver, and kidney function, and did not take psychotropic drugs and antidepressants in the near future. They have no history of traumatic brain injury, neurological diseases and alcohol abuse, no severe hearing and visual impairment. All of them were assessed with MMSE screening scale before operation and had no cognitive dysfunction. The characteristics of participants are shown in Table 1. Then they were randomly assigned into 2 groups using a random number table: control group (Group C) and nimodipine group (Group N). The sample size of the study was calculated according to previous studies and was based on a pilot study.

**Table 1:**
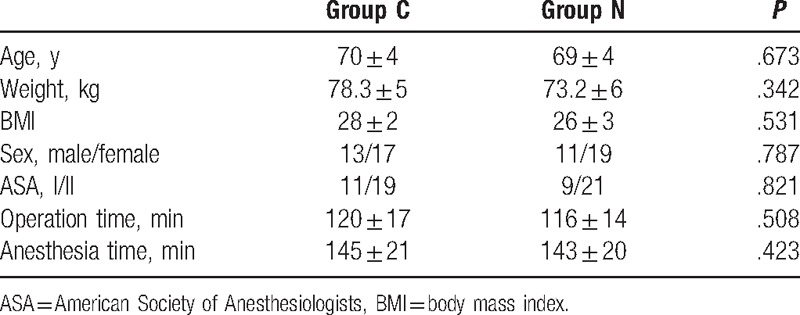
Basic demographic data and surgery/anesthesia-related information (n = 30).

### 2.3 Intervention methods

**Figure 1:**
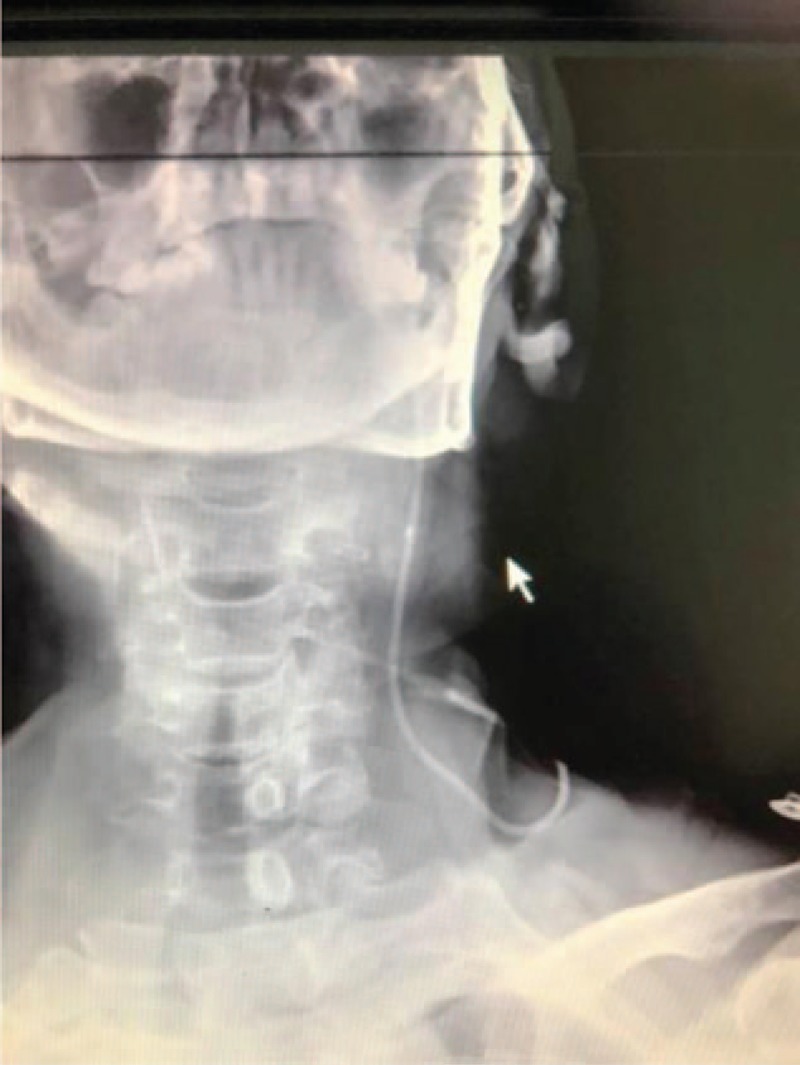
X-ray image of patient receiving ultrasound-guided retrograde puncture of internal jugular vein.

After entering the operating room, electrocardiogram, pulse oxygen saturation, respiratory rate, PetCO_2_, bispectral index (BIS), and invasive blood pressure were recorded. As shown in Fig. 1, all patients received ultrasound-guided retrograde puncture of internal jugular vein, then 12 to 14 cm of catheter was inserted into the deep vein catheter to jugular bulb using Seldinger method and the location of the catheter was confirmed by X-ray.

In Group N, patients were venously and persistently injected with 7.5 μg/(kg × h) nimodipine (Bayer Schering Pharma AG, Leverkusen, Germany) and patients were injected with equivalent normal saline in Group C. Anesthesia was induced by intravenous injection of midazolam 0.05 to 0.2 mg/kg, 0.3 mg/kg etomidate, 0.1 to 2 μg/kg sulfentanil, and 0.15 mg/kg cisatracurium. Mechanical ventilation was performed after establishing endotracheal intubation. Ventilation frequency was set to 12 times per minute, inspiratory expiratory ratio to 1.0:1.5, inhaled oxygen concentration to 100%, oxygen flow to 2 L per minute, and PaCO_2_ was maintained within the physiologic limits (35–45 mm Hg). Anesthesia was maintained with continuous infusion of propofol 4 to 6 mg/(kg × h) and remifentanil 0.1 to 0.3* *μg/(kg × min), keeping the BIS value of 40 to 60, blood pressure fluctuation amplitude did not exceed 20% of the base value. Nimodipine, normal saline, remifentanil, and propofol were stopped when the skin was cut off. Tracheal intubation was removed when patients regained consciousness, tidal volume was 8 mL/kg and all hemodynamic parameters such as heart rate, blood pressure returned to normal level.

### 2.4 Data collection and index detection

At 0 minute before injection (T_1_), 0 minute after tracheal intubation (T_2_), 1 hour after skin incision (T_3_), and surgery completed (T_4_), 2 mL blood samples were taken from the radial artery and jugular bulb for blood gas analysis by automatic blood gas analyzer (RADUOMETER ABL800, Denmark). Hb, arterial oxygen saturation (SaO_2_) and arterial partial pressure of oxygen (PaO_2_), jugular bulb oxygen saturation (CjvO_2_), and jugular bulb oxygen pressure (PjvO_2_) were recorded. Arterial oxygen content (CaO_2_), CjvO_2_, arterial jugular bulb oxygen content difference (Ca-jvO_2_), and extracting rate of oxygen (CERO_2_) were calculated by Fick formula:

CaO_2_ (mL/L) = Hb × 1.36 × SaO_2_ + 0.0031 × PaO2

CjvO_2_ (mL/L) = Hb × 1.36 × SjvO_2_ + 0.0031 × PjvO_2_

Ca-jvO_2_ (mL/L) = CaO_2_-CjvO_2_

CERO_2_(%) = Ca-jvO_2_/ CaO_2_ × 100%

At the same time point, 4 mL blood samples were taken from the jugular bulb. Then the blood samples were centrifuged separately and the supernatant was collected and stored at −70°C. Serum S100β and Neuron specific enolase (GFAP) were measured with a commercially available S100β ELISA kit (Merck-Millipore, Leverkusen, Germany) and GFAP ELISA kit (NeoMarkers, Leverkusen).

Incidence of postoperative delirium was assessed by nursing delirium screening score (Nu-DESC)^[10]^ in 1 to 7 days after surgery: each symptom was scored 0 to 2 on the basis of the severity, the sum of scores of each time gets a total score, the highest score is 10 points, patients scoring more than 2 were diagnosed with delirium.

### 2.5 Statistical analysis

All data were analyzed by SPSS (version 21.0 for Windows, SPSS Inc, Chicago, IL). Measurement data of normal distribution were reported as the mean ± SD. Comparisons between 2 groups were performed with *t* test; comparisons among different were done with one way analysis of variance. Two-tailed probability value of *P* < .05 was considered statistically significant.

## 3 Results

### 3.1 Demographic data and surgery/anesthesia-related information

Sixty patients were enrolled and assigned into 2 groups (n = 30, 7 patients were lost because of noncooperation and 4 patients were lost by not receiving operation). As shown in Table 1, patients from 2 groups had comparable demographic and surgery/anesthesia-related variables, including age, weight, BMI, sex, ASA class, operation time, and anesthesia time.

### 3.2 Cerebral oxygen metabolism-related indicators

All the cerebral oxygen metabolism-related indicators fluctuationed in the normal range. There were no significant differences in CaO_2_ (t = 0.702, *P* = .445), CjvO_2_ (t = 0.854, *P* = .40), Ca-jvO_2_ (t = 0.736, *P* = .457), and CERO_2_ (t = 0.878, *P* = .394) at T_1_ in 2 groups. There were no significant differences in all time points during operation in CaO_2_ (Group C T_2_:t = 0.687, *P* = .51; T_3_:t = 0.592, *P* = .54; T_4_:t = 1.308, *P* = .20; Group N T_2_:t = 0.846, *P* = .41; T_3_:t = 0.787, *P* = .46; T_4_:t = 1.628, *P* = .11) and CjvO_2_ in Group C (T_2_:t = 0.849, *P* = .409; T_3_:t = 1.383, *P* = .164; T_4_:t = 1.678, *P* = .112). Compared with T_1_, CjvO_2_ increased in Group N at T_3_ (t = 2.137, *P* = .048) and T_4_ (t = 2.454, *P* = .021) and Ca-jvO_2_ (Group C T_3_:t = 2.376, *P* = .028; T_4_:t = 2.715, *P* = .011; Group N T_3_:t = 2.483, *P* = .019; T_4_:t = 3.041, *P* = .005) and CERO_2_ (Group C T_3_:t = 2.109, *P* = .044; T_4_:t = 2.573, *P* = .017; Group N T_3_:t = 2.334, *P* = .018; T_4_:t = 2.462, *P* = .02) decreased at T_3_ and T_4_. Compared with Group C, Ca-jvO_2_ and CERO_2_ decreased at T_3_ (Ca-jvO_2_ t = 2.356, *P* = .022; CERO_2_ t = 2.733, *P* = .019) and T_4_ (Ca-jvO_2_ t = 2.981, *P* = .008; CERO_2_ t = 2.109, *P* = .048) in Group N (Table 2).

**Table 2:**
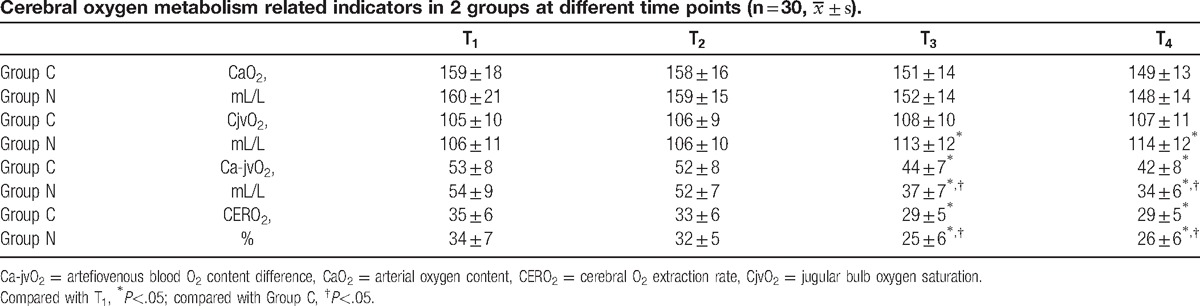


### 3.3 Cerebral damage-related indicators

**Figure 2:**
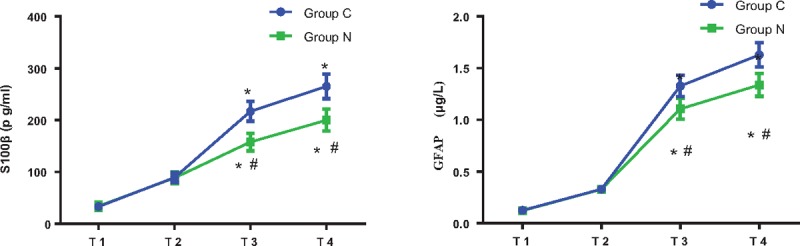
Levels of S100β and GFAP in 2 groups at different time points. T_1_: 0 minute before injection; T_2_: 0 minute after tracheal intubation; T_3_: 1 hour after skin incision; T_4_: surgery completed. Compared with T_1_, ^∗^*P*<.05; compared with Group C, ^#^*P*<.05.

There was no significant difference in S100β and GFAP between 2 groups at T_1_ (S100β: t = 0.874, *P* = .389; GFAP: t = 0.965, *P* = .317) and T_2_(S100β: t = 0. 937, *P* = .315; GFAP: t = 1.116, *P* = .275). Compared with T_1_, S100β (Group C: T_3_:t = 2.371, *P* = .024; T_4_:t = 2.462, *P* = .02; Group N: T_3_:t = 2.107, *P* = .043; T_4_:t = 2.484, *P* = .019) and GFAP (Group C: T_3_:t = 2.756, *P* = .01; T_4_:t = 2.631, *P* = .017; Group N: T_3_:t = 2.879, *P* = .009; T_4_:t = 2.747, *P* = .011) levels were elevated in the 2 groups at T_3–4_ (*P*<.05). Compared with Group C, S100β (T_3_:t = 3.038, *P* = .005; T_4_:t = 2.741, *P* = .009) and GFAP (T_3_:t = 2.546, *P* = .019; T_4_:t = 2.973, *P* = .008) levels were decreased in Group N at T_3–4_ (*P*<.05, Fig. 2).

### 3.4 Incidence of postoperative delirium

**Figure 3:**
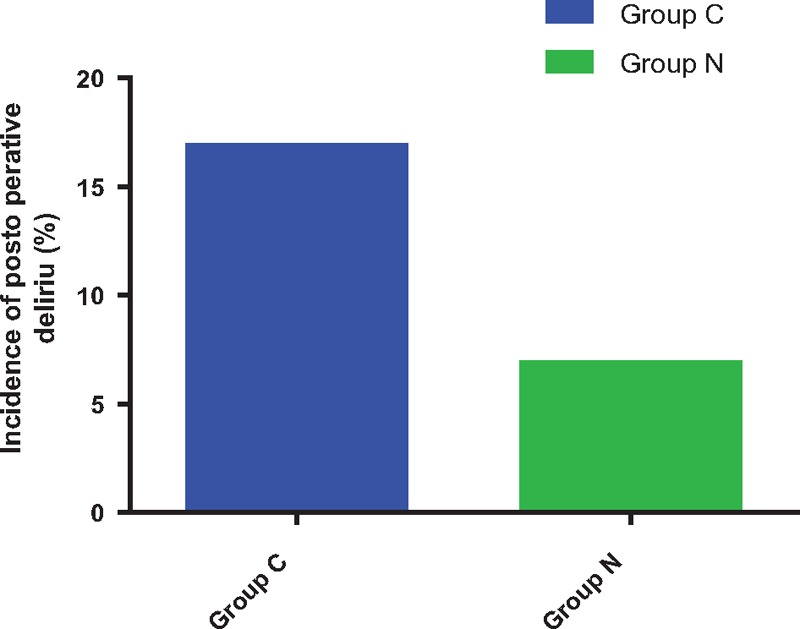
Incidence of postoperative delirium. Compared with Group C, ^∗^*P*<.05.

As shown in Fig. 3, compared with Group C, incidence of postoperative delirium (t = 2.537, *P* = .017) decreased in Group N after surgery.

## 4 Discussion

Our previous study showed pretreatment with nimodpine was able to attenuate the POCD in aged rats.^[9]^ This study was undertaken to investigate the effects of nimodipine on postoperative delirium in elderly under general anesthesia. Our results further showed nimodipine can reduce the development of postoperative delirium in elderly patients under general anesthesia, the reduction of brain injury may be involved in the mechanism.

Due to extrusion of inferior vena cava caused by prone position, rich venous plexus in spinal canal, large operation range and parenchyma bleeding and large amount of blood loss, the balance of important organs would be interfered during spinal surgery.^[11]^ Cerebral oxygen metabolism can reflect the matching relationship between oxygen supply and oxygen consumption in the whole brain, and the state of cerebral circulation more accurately. It is considered to be an important basis for early detection of cerebral ischemia and hypoxia. Studies have shown that the incidence of postoperative cognitive dysfunction in elderly patients with noncardiac surgery is related to the abnormal cerebral oxygen metabolism during operation.^[12]^ Ca-jvO_2_ and CERO_2_ are the important index for monitoring cerebral oxygen metabolism. They can directly reflect the degree of uptake or reduce of oxygen in brain. Declining of them indicates cerebral oxygen metabolism rate decreases, cerebral blood flow is sufficient to meet the brain oxygen consumption. The jugular bulb blood flow directly from brain tissue, oxygen saturation, and oxygen partial pressure of internal carotid are the same with the radial artery, so we used jugular bulb blood instead of cerebral venous blood, used oxygen saturation and oxygen partial pressure of radial artery instead of carotid artery oxygen saturation and oxygen partial pressure. Results confirmed nimodipine can improve cerebral oxygen metabolism of aged surgical patients under general anesthesia.

S100β and GFAP are the important neurological biomarkers of cerebral damage.^[13]^ S100β that secreted by astrocytes and oligodendrocytes in the central nervous system is a kind of small molecular weight calcium binding protein. After brain injury (mild brain injury, ischemic brain injury), the level of S100βin serum was significantly increased accompanied by a certain regularity of time, and it is regarded as one of the most important markers of brain damage. GFAP is a kind of intermediate filament protein, which exists in the form of monomer, mainly distributed in the astrocytes of the central nervous system and participates in the formation of cytoskeleton and maintains its tensile strength. Previous study found that content of S100β and GFAP in the brain is closely related to the occurrence of postoperative delirium.^[14,15]^ In this study, our data indicated that nimodipine can decrease the level of S100β and GFAP and reduce cerebral damage of aged surgical patients under general anesthesia.

The present study has several limitations. First, the number of patients is relatively small. Second, we only studied elderly patients undergoing spine surgery; the effects of nimodipine on postoperative delirium in elderly patients receiving other surgeries need our further study.

In conclusion, nimodipine can reduce the development of postoperative delirium in elderly patients under general anesthesia, the reduction of brain injury, and improvement of cerebral oxygen metabolism may be involved in the mechanism.
